# Identification of Nitrogen Use Efficiency Genes in Barley: Searching for QTLs Controlling Complex Physiological Traits

**DOI:** 10.3389/fpls.2016.01587

**Published:** 2016-10-21

**Authors:** Mei Han, Julia Wong, Tao Su, Perrin H. Beatty, Allen G. Good

**Affiliations:** ^1^Department of Biological Sciences, University of AlbertaEdmonton, AB, Canada; ^2^Co-Innovation Center for Sustainable Forestry in Southern China, College of Biology and the Environment, Nanjing Forestry UniversityNanjing, China

**Keywords:** nitrogen use efficiency (NUE), QTL, phenotyping, MAS, barley

## Abstract

Over the past half century, the use of nitrogen (N) fertilizers has markedly increased crop yields, but with considerable negative effects on the environment and human health. Consequently, there has been a strong push to reduce the amount of N fertilizer used by maximizing the nitrogen use efficiency (NUE) of crops. One approach would be to use classical genetics to improve the NUE of a crop plant. This involves both conventional breeding and quantitative trait loci (QTL) mapping in combination with marker-assisted selection (MAS) to track key regions of the chromosome that segregate for NUE. To achieve this goal, one of initial steps is to characterize the NUE-associated genes, then use the profiles of specific genes to combine plant physiology and genetics to improve plant performance. In this study, on the basis of genetic homology and expression analysis, barley candidate genes from a variety of families that exhibited potential roles in enhancing NUE were identified and mapped. We then performed an analysis of QTLs associated with NUE in field trials and further analyzed their map-location data to narrow the search for these candidate genes. These results provide a novel insight on the identification of NUE genes and for the future prospects, will lead to a more thorough understanding of physiological significances of the diverse gene families that may be associated with NUE in barley.

## Introduction

Global food production has been increased markedly as a result of several major factors during the past half century. The first of these was the use of synthetic fertilizers after World War II, followed by the “Green Revolution” in the 1960s. The advent of modern biotechnology in the 1990s introduced genetically modified organisms (GMOs), while innovations in crop management and agricultural mechanization have also been important drivers of increases in productivity. Interestingly, the first two factors driving these increases in yield are both related to nitrogen, which is one of the fundamental elements required for plant growth.

Nitrogen (N) absorption by plants is comprised of three major steps: uptake, assimilation and remobilization. NUE is the product of N uptake efficiency (NUpE) and N utilization efficiency (NUtE; Good et al., 2004). Increased NUE uptake usually results in increased above-ground biomass, seed production, grain protein, and yield in crops (Masclaux-Daubresse et al., 2010). Fixed nitrogen, which can be provided by soil microbes or as synthetic fertilizer, is taken up as nitrate (${\text{NO}}_{3}^{-}$) or ammonium (${\text{NH}}_{4}^{+}$) and utilized for multiple metabolic processes, including amino acid synthesis as well as signaling and storage molecules (Stitt et al., 2002). Although the use of synthetic N fertilizers on crops significantly improves performance for yield-related traits, most crop plants absorb only 30–50% of the N fertilizer applied, depending on the soil, the environment, and the plant population (Tilman et al., 2002). More than half of the nutrients applied are not used by the plant and are lost into the environment, giving rise to profound impacts ranging from air and water contamination to the undermining of ecosystems (Wuebbles, 2009; Ng et al., 2016). The total crop yields in many intensive farming systems have failed to improve in proportion to the application of chemical fertilizers, leading to low NUE and more serious environmental N pollution (Shen et al., 2013). A recent report revealed that between 1960 and 2008, 24–39% of crop growing areas for maize, rice, wheat and soybean have had yields that either not improved, have stagnated, or collapsed (Ray et al., 2012). These data underscore the challenges and potentials of increasing global food demands while implementing new strategies to improve crop yield, and concurrently reducing N inputs in the coming years.

Theoretically, two approaches are applicable to improve NUE in crops: (1) A traditional breeding strategy combined with MAS, and (2) a transgenic approach, targeting specific NUE-associated genes for the genetic engineering of the plant. The latter has been recently reviewed (Good and Beatty, 2011; McAllister et al., 2012), and will not be considered further. Hitherto, despite significant investments in this area of research, no organizations released a crop variety that has been shown to be more nutrient efficient. Although traditional genetic approaches to improve NUE have been widely attempted for the major cereals (i.e., maize, rice, wheat and soybean), only limited studies have been performed to extensively explore the candidate genes associated with NUE and their relationships with NUE phenotypes.

Barley (*Hordeum vulgare*) is one of the earliest domesticated crops and the current interests in barley as the healthy food and malting component have been increasing. As the extensive physiological information available on N uptake and transport, barley has become an important model species for *Triticeae* genomics. In contrast with wheat, barley has more advantages of a less complex genome (diploid), the integrated genome sequence database, and the focus of a large international collaborative effort to develop new genomic technologies (Mayer et al., 2012). Here, based on those that have been experimentally shown to be involved in NUE, a large number of candidate genes that may be responsible for NUE phenotypes were characterized and mapped. We then performed an analysis of genetic locations between NUE genes and independent mapping studies reported QTLs related to NUE components. Our main objective was to provide initial information of NUE-associated genes and their potential relevance to NUE phenotypes in barley. In a long term, specific genes for NUE will be targeted for investigating physiological roles in NUE regulation as well as the improvement of NUE in barley breeding. A comparison of these QTLs for NUE with a number of the characterized NUE genes illustrates the challenges in identifying candidate genes associated with natural variation for NUE traits.

## Materials and methods

### Gene analysis and genetic map location

The list of NUE genes is based on several of our recent reviews (Good and Beatty, 2011; McAllister et al., 2012). The logic for the selection of each of these genes is discussed below in Results. The genetic locations of all candidate NUE genes were mapped using the “Morex” × “Barke” population or, in cases where a map location had not been assigned for a particular gene, the Oregon-Wolfe population was used instead. The MSU Rice Genome Annotation Browser (http://rice.plantbiology.msu.edu/) was used to obtain the protein sequences of the candidate genes from rice (**Table 2** and Table S1). The gene and protein sequences were collected to seed a BLAST search against the Barley WGS Morex Assembly version 3, using the default settings of the respective websites for BLAST searches (Mayer et al., 2012). The accession numbers (ID or MLOC_#) for the gene sequences in barley were obtained from the IPK Barley Blast Server (http://webblast.ipk-gatersleben.de/barley) and the James Hutton Institute (http://ics.hutton.ac.uk). The protein sequence alignments between characterized rice homologs and barley candidate genes were manually checked using M-Coffee (http://www.tcoffee.org/Projects/mcoffee/) and were further validated by a reciprocal BLAST search between the rice and barley genomes. The barley candidate genes ID, their genetic locations (cM), number of gene model (http://plants.ensembl.org/index.html), the presence of a full length cDNA (fl cDNA), and other relevant information are given in **Table 2**. The genetic location of each locus was based on the “Morex” × “Barke” recombinant inbred lines (RIL) mapping population, unless otherwise indicated.

### Expression analysis

Expression analyses for a subset of barley NUE candidate genes (glutamate-pyruvate transaminase (GPT), glutamate glyoxylate aminotransferase (GGT), high-affinity nitrate transporters (NRT2), and the associated partner protein (NAR2) families) were performed using morexGenes-Barley RNA-seq that is accessible from the James Hutton Institute (https://ics.hutton.ac.uk/morexGenes/blast_page.html). This database contains gene global expression patterns in barley, including eight tissues from the cultivar Morex, with three replicates assayed per tissue (Mayer et al., 2012). The tissues examined were: germinating embryo (EMB, 4 days after germination), young leaf tissue (LEA, from a 10 cm high plant), young root tissue (ROO, from a 10 cm high plant), developing inflorescence (INF1, 5 mm-long inflorescence and INF2, 10–15 mm-long inflorescence), the third internode (NOD, 42-day-old plants) and two time points for the developing caryopsis (CAR5, 5 days after anthesis and CAR15, 15 days after anthesis). The data were presented in FPKM (fragments per kilobase of exon per million fragments mapped) expression values. Additional data of expression analysis was conducted using microarray that is accessible from BarleyBase (http://www.plexdb.org/plex.php?database=Barley). Similar tissues (except for IMM INF, immature inflorescence; PIS, pistil; CAR16, 16 days after anthesis) of Morex were used for microarray analysis with three replicates assayed per tissue. The detailed information of probe set was described in Table S2. Heat map for the microarray data was constructed by the online program CIMminer (http://discover.nci.nih.gov/cimminer/home.do).

### Multiple sequence alignment and phylogenetic tree construction

A subset of the gene families (GPT, GGT, NRT2, and NAR2) were analyzed in more detail. The protein sequences for the members of specific gene families were aligned using the MUSCLE algorithm of the Molecular Evolutionary Genetics Analysis 6.0 (MEGA6) software (http://www.megasoftware.net/; Tamura et al., 2013). A phylogenetic tree was constructed using neighbor-joining method from protein sequences the GPT, GGT, NRT2, and NAR2 family members. Statistical support was given as consensus bootstrap values from 5000 replicate tests for each tree. The phylogenetic trees are drawn to scale, with branch lengths in the same units as those of the evolutionary distances used to infer the phylogenetic tree.

### Field trials selection

Independent QTLs studies from 10 publications between 2003 and 2015 were chosen to perform further analyses. A diverse set of markers were used in these studies to map the loci for specific NUE-related traits (Table S4). Two criteria were set up and applied to assess the experimental data used for the QTL analysis. First, these experiments were evaluated on the basis of whether they were laboratory experiments or field trials. Only field trial studies [an exception goes for Kindu et al. (2014), which directly showed the mapped NUE traits] were accepted and ideally, the experiments were run for more than one season. Second, specific NUE-related agronomic traits were measured, including grain protein content (GPC), grain yield (YLD), grain weight (GW). NUE traits with the N remobilization efficiency (ΔN), N harvest index (NHI), and grain N content (GN) were also included. The detailed definitions for selected traits are provided in the legend for **Table 3**.

### Markers normalization and projection of candidate NUE genes

The selected publications used markers that were usually normalized on the consensus map on the basis of GrainGenes (http://wheat.pw.usda.gov/cgi-bin/graingenes/browse.cgi?class=marker). The dataset of SNP markers used on barley consensus genetic map are described in Table S5 and Datasheet 1. The SNP markers were used to reconstruct barley consensus genetic map based on a “Morex” × “Barke” population, which originally contains 2994 SNP loci mapped to 1163 unique positions and spans 1137.3 cM with an average density of one marker bin per 0.99 cM (Muñoz-Amatriaín et al., 2011, 2014). More detailed sequence information of markers is accessible from HarvEST (http://harvest.ucr.edu/). The reconstruction of barley genetic map was conducted by MapDisto 1.7.7.011 software (http://mapdisto.free.fr/Download_Soft/). In addition to a subset of markers shown, the candidate genes for NUE were placed on the consensus map based their genetic locations (cM; **Table 2**).

## Results

### Identification and nomenclature of barley candidate NUE genes

To select barley NUE candidate genes, we performed a homologous BLAST in barley genome based on NUE genes in rice. The list of identified candidate genes was divided into six categories based on their specific physiological functions and potential roles in affecting NUE in plants: signaling, amino acid biosynthesis, N assimilation, transcription factors (TFs), transporters, and other uncategorized genes (Tables 1, 2). The BLAST searches retrieved a large number of homologous candidate gene members for each gene family queried. In total, 113 barley genes were identified (Table 1). The name associated with the gene members of a particular family (e.g., GPT) was based on the chromosome designation and an increasing gene number as one moved down the chromosome (e.g., *AlaAT 2-1*).

**Table 1:** **A list of genes with potential roles in improving NUE in plants**.

**Name**	**Gene family**	**Host**	**Effect on phenotype related with NUE**	**References**
**SIGNALING GENES**				
*DEP1*	G-protein γ subunit	Rice	N uptake, assimilation; grain yield increased at moderate levels of N input	Sun et al., 2014
*SMG1*	Mitogen-activate kinase kinase	Rice	Impact on grain size and panicle density	Duan et al., 2014
*SnRK*	SNF1-related kinase	Tomato	Higher NUpE in overexpressing plant	Wang et al., 2012b
*ENOD*	Early nodulin like protein	Rice	Increased total amino acids and N as well as dry biomass and seed yield	Bi et al., 2009
**AMINO ACID BIOSYNTHESIS GENES**				
*AlaAT*	Alanine aminotransferase	Rice	Increased seed yield both in laboratory and field under low N input	Shrawat et al., 2008
*ASN*	Asparagine synthetase	*Arabidopsis*	N content and seed yield at high N and low N input	Lam et al., 2003
*aspAT/ASP*	Aspartate aminotransferase	*Arabidopsis*	Increased AspAT activity and PEPc activity	Ivanov et al., 2012
*ASNase*	Asparaginase	Rice	N utilization from regulation of maize asparagine cycling and homeostasis	Zhou et al., 2009
*gdhA/GDH*	NADP-dependent glutamate dehydrogenase	Rice	Several folds higher levels of free amino acids including glutamate	Abiko et al., 2010
*GS*	Glutamine synthetase	Rice	NUE increased under high N condition	Brauer et al., 2011
*GOGAT*	Glutamate synthase	Rice	Improved grain filling, total nitrogen content, and dry weight	Tamura et al., 2011
**N ASSIMILATION GENES**				
*NR*	Nitrate reductase	Tobacco	Nitrate content increased in leaves and high NO emission	Lea et al., 2006
*NiR*	Ferredoxin-Nitrite reductase	*Arabidopsis*	NO^2−^ assimilation increased	Takahashi et al., 2001
**TRANSCRIPTIONAL FACTORS**				
*DOF*	DNA-binding One Zinc Finger	Rice	Increased growth, N assimilation, and enhanced grain production	Li et al., 2013
*SAT1*	bHLH transcription factor	Soybean	Nodulization to improve N fixation and NH^4+^ transport	Chiasson et al., 2014
*NFY*	Nuclear factor Y	Rice	Increased drought and salinity tolerance and grain yield	Chen et al., 2015
*NAC*	NAM, ATAF1,2, and CUC2	Wheat	Enhanced drought resistance; senescence, nutrient remobilization, and grain protein content	Uauy et al., 2006
*APO*	F-box protein	Rice	Grain yield improved per plant	Terao et al., 2010
**TRANSPORTER GENES**				
*NRT*	Nitrate transporter	*Arabidopsis*	Nitrate content and dry weight increased in shoots	Léran et al., 2015a
*AMT*	Ammonium transporter	Rice	Increased ammonium uptake and reduced dry weight under high Am	Yuan et al., 2007
*LHT*	Lysine histidine transporter	*Arabidopsis*	Improved plant growth under low N condition	Hirner et al., 2006
*STP13*	Hexose transporter	*Arabidopsis*	Growth, biomass, and N use increased by application of exogenous sugar	Schofield et al., 2009
**OTHER GENES**				
*CKX*	Cytokinin oxidase/dehydrogenase	Rice	More panicles and grain numbers	Ashikari et al., 2005
*IPT*	Isopentenyl transferase	*Arabidopsis*	Delayed senescence when grown under low N input	Rubio-Wilhelmi et al., 2011
*CIN*	Cell wall invertase	Rice	Grain weight and seed filling impacted	Wang et al., 2008
*SGR*	Stay-green protein	Rice	Delays senescence, LHC II is stable in SGR mutant rice	Park et al., 2007
*FNR*	Ferredoxin NADP(H) reductase	Rice	Improved root growth, ear size and seed weight	Hanke et al., 2008

**Table 2:** **Candidate genes involved in NUE in rice and barley**.

**NUE genes**	**Barley (*Hordeum vulgare*)**							**Rice (*Oryza sativa*)**		
	**Candidate gene**	**Chr**	**Morex contig**	**MxBk (cM)**	**MLOC**	**Gene models**	**fl cDNA**	**Candidate gene**	**Chr**	**Locus name**
**SIGNALING GENES**										
Heterotrimeric G-Protein	*HvDEP1*	5H	contig_37321	52.29	MLOC_52150^L^	1	na	*qNGR9/DEP1*	9	LOC_Os09g26999
	*HvRGA1*	7H	contig_52745	9.06	MLOC_67224	8	Y	*D1/OsRGA1*	1	LOC_Os05g26890
	*HvRGB1*	4H	contig_65187	11.38	MLOC_74118	2	N	*OsRGB1*	3	LOC_Os03g46640
Mitogen-activate kinase kinase (MKK)	*HvSMG1*	6H	contig_1564374	78.4	MLOC_12915	2	Y	*OsSMG1*	9	LOC_Os09g28520
	*HvSMG2*	5H	contig_134755	68.3	MLOC_4150	2	N	*OsSMG2*	2	LOC_Os02g52490
Sucrose non-fermenting-1 related kinases (SnRK)	*HvPKABA1*	2H	contig_1561710	114.66	MLOC_11726	5	Y	*OsSAPK1*	3	LOC_Os03g27280
	*HvPKABA2*	2H	contig_5609	53.68	MLOC_69212	1	Y	*OsSAPK2*	7	LOC_Os07g42940
	*HvPKABA3*	4H	contig_160302	51.4	MLOC_22145	4	Y	*OsSAPK3*	3	LOC_Os03g55600
	*HvPKABA4*	5H	contig_127028	43.96	MLOC_3013	6	Y	*OsSAPK4*	1	LOC_Os01g64970
	*HvPKABA5*	2H	contig_46940	58.64	MLOC_62759	4	Y	*OsSAPK5*	2	LOC_Os02g34600
	*HvPKABA6*	5H	contig_160473	129.93	MLOC_22271	4	Y	*OsSAPK6*	10	LOC_Os10g41490
	*HvPKABA7*	2H	contig_1565788	148.16	MLOC_13479	2	Y	*OsSAPK7*	4	LOC_Os04g35240
	*HvPKABA8*	3H	contig_47971	86.33	MLOC_63787	3	N	*OsSAPK8*	3	LOC_Os03g41460
	*HvPKABA9*	1H	contig_99735	86.54	MLOC_82073	5	N	*OsSAPK9*	12	LOC_Os12g39630
Early nodulin like protein	*HvEND93-1*	7H	contig_1635653	23.8	MLOC_24054	1	Y	OsEND93-1^*^	6	LOC_Os06g05010
	*HvEND93-2*	7H	contig_45347	43.59	MLOC_61290	1	Y	*OsEND93-2*	6	LOC_Os06g04990
	*HvEND93-3*	6H	contig_2552301	55.52	MLOC_39111	2	N	*OsEND93-3*	6	LOC_Os06g05020
**AMINO ACID BIOSYNTHESIS GENES**										
Glutamic-pyruvate transaminase (alanine aminotransferase; GPT)	*HvAlaAT1-1*	1H	contig_51312	46.32	MLOC_66262^L^	1	na	*OsAlaAT10-1*	10	LOC_Os10g25130
	*HvAlaAT2-1*	2H	contig_37898	54.25	MLOC_52901	1	Y	*OsAlaAT10-2*	10	LOC_Os10g25140
	*HvAlaAT2-2*	2H	contig_57179	58.78	MLOC_69931	3	Y	*OsAlaAT2*	9	LOC_Os09g26380
	*HvAlaAT5-1*	5H	contig_138706	42.15	MLOC_7150	9	Y	*OsAlaAT3-1*	7	LOC_Os07g01760
	*HvAlaAT5-2*	5H	contig_51539	49.89	MLOC_66427	5	Y	*OsAlaAT3-2*	7	LOC_Os07g42600
								*OsAlaAT4*	3	LOC_Os03g08530
Glutamate glyoxylate aminotransferase (GGT)	*HvGGT1*	1H	contig_45148	76.84	MLOC_57145	2	Y	*OsGGT1*	5	LOC_Os05g39770
	*HvGGT2*	4H	contig_1577122	81.6	MLOC_17573	3	Y	*OsGGT2*	3	LOC_Os03g07570
								*OsGGT3*	3	LOC_Os03g21960
Asparagine synthetase	*HvASN1*	4H	contig_274144	54.82	MLOC_44080	1	Y	*OsASN1*	3	LOC_Os03g18130
	*HvASN4*	5H	contig_47260	46.46	MLOC_63089	13	Y	*OsASN2*	6	LOC_Os06g15420
Asparaginase	*HvASNase1*	2H	contig_48445	91.01	MLOC_64169	14	Y	*OsASNase1*	4	LOC_Os04g46370
	*HvASNase2*	2H	contig_51188	142.63	MLOC_66166	12	Y	*OsASNase2*	4	LOC_Os04g58600
Aspartate aminotransferase	*HvASP1*	6H	contig_1573332	100.99	MLOC_16420	1	Y	*OsASP1*	2	LOC_Os02g55420
	*HvASP2*	1H	contig_156882	86.54	MLOC_14736	5	Y	*OsASP2*	6	LOC_Os06g35540
	*HvASP3*	7H	contig_2547742	76.47	MLOC_37080	3	Y	*OsASP3*	2	LOC_Os02g14110
	*HvASP4*	3H	contig_1566402	63.5	MLOC_13742	1	Y	*OsASP4*	1	LOC_Os01g55540
	*HvASP5*	6H	contig_90524	10.27	MLOC_80438	1	Y	*OsASP5*	1	LOC_Os01g65090
	*HvASP6*	5H	contig_40146	68.3	MLOC_55643	1	Y	*OsASP6*	10	LOC_Os10g34350
	*HvASP7*	3H	contig_159523	45.82	MLOC_21451	2	Y	*OsASP7*	9	LOC_Os09g28050
Asparagine synthase	*HvAS*	5H	contig_9597	42.99	MLOC_81375	7	N	*OsAS*	12	LOC_Os12g38630
Glutamate dehydrogenase NAD(P)H	*HvGDH1*	5H	contig_55763	139.24	MLOC_69020	4	Y	*OsGDH1*	3	LOC_Os03g58040
	*HvGDH2*	3H	contig_499299	51.35	MLOC_65227	6	N	*OsGDH2*	4	LOC_Os04g45970
	*HvGDH3*	2H	contig_79282	81.8	MLOC_78233	3	Y	*OsGDH3*	2	LOC_Os02g43470
	*HvGDH4*	3H	contig_2547948	52.03	MLOC_37189	1	N	*OsGDH4*	1	LOC_Os01g37760
Glutamine synthetase	*HvGS1*	6H	contig_1562081	68.7	MLOC_11890	8	Y	*OsGS1*	2	LOC_Os02g50240
	*HvGS2*	4H	contig_1569958	60.69	MLOC_15134^L^	1	na	*OsGS2*	3	LOC_Os03g12290
	*HvGS3*	2H	contig_38845	120.04	MLOC_54057	9	Y	*OsGS3*	3	LOC_Os03g50490
	*HvGS4*	4H	contig_46131	27.8	MLOC_62034^L^	3	na	*OsGS4*	4	LOC_Os04g56400
	*HvGS5*	4H	contig_1573852	59.49	MLOC_16584^L^	1	na			
Glutamate synthase (NADPH/Ferredoxin)	*HvGOGAT1*	3H	contig_1566054	51.62	MLOC_13604	3	N	*GOGAT1*	1	LOC_Os01g48960
	*HvGOGAT2*	2H	contig_5871	50.04	MLOC_70866	3	Y	*GOGAT2*	7	LOC_Os07g46460
								*GOGAT3*	5	LOC_Os05g48200
Glycolate oxidase (GOX)	*HvGOX1*	2H	contig_1572170	58.05	MLOC_16035	1	Y	*OsGOX1*	3	LOC_Os03g57220
	*HvGOX2*	2H	contig_65448	58.64	MLOC_74253^L^	5	na	*OsGOX2*	4	LOC_Os04g53210
	*HvGOX3*	5H	contig_6695	136.59	MLOC_75010	4	Y	*OsGOX3*	4	LOC_Os04g53214
	*HvGOX4*	2H	contig_52591	54.32	MLOC_67111^L^	8	na	*OsGOX4*	7	LOC_Os07g05820
	*HvGOX5*	na	contig_46080	na	MLOC_61991	3	Y	*OsGOX5*	7	LOC_Os07g42440
**GENES FOR N ASSIMILATION**										
Nitrate reductase	*HvNR1*	6H	contig_136596	82.36	MLOC_5716	2	Y	*OsNR1*	8	LOC_Os08g36500
	*HvNR2*	6H	contig_44311	10.27	MLOC_60358	1	Y	*OsNR2*	2	LOC_Os02g53130
								*OsNR3*	8	LOC_Os08g36480
								*OsNR4*	10	LOC_Os10g17780
Ferredoxin-nitrite reductase	*HvNiR1*	6H	contig_273133	87.32	MLOC_43860	2	N	*OsNiR1*	1	LOC_Os01g25484
	*HvNiR2*	2H	contig_181042	43.97	MLOC_27159	1	N	*OsNiR2*	1	LOC_Os01g25520
								*OsNiR3*	2	LOC_Os02g52730
								*OsNiR4*	5	LOC_Os05g42350
**TRANSCRIPTIONAL FACTORS**										
DNA-binding One Zinc Finger (DOF)	*HvDOF1*	5H	contig_327	75.88	MLOC_48629	1	Y	*DOF1*	8	LOC_Os08g38220
	*HvDOF2*	2H	contig_160092	58.64	MLOC_21982	1	Y	*DOF2*	12	LOC_Os12g39990
	*HvDOF3*	5H	contig_2548810	130.35	MLOC_37654	1	N	*DOF3*	3	LOC_Os03g55610
	*HvDOF4*	1H	contig_157123	17.28	MLOC_15655	1	N	*DOF4*	9	LOC_Os09g29960
	*HvDOF5*	7H	contig_49081	69.56	MLOC_64612	2	Y	*DOF5*	5	LOC_Os05g02150
Nuclear factor Y (NFY)	*HvNF-YB2.1*	1H	contig_2547450	85.64	MLOC_36879	7	N	*OsNF-YB2.1*	5	LOC_Os05g38820
	*HvNF-YB2.2*	3H	contig_6163	98.65	MLOC_72428	5	Y	*OsNF-YB2.2*	1	LOC_Os01g61810
	*HvNF-YB2.3*	2H	contig_42088	67.49	MLOC_57782	1	N	*OsNF-YB2.3*	2	LOC_Os03g29970
bHLH transcriptional factor	*HvHLHm1*	4H	contig_40514	59.63	MLOC_56065	3	N	*OsHLHm1*	3	LOC_Os03g12760
	*HvHLHm2*	4H	contig_49250	36.35	MLOC_64735	2	Y	*OsHLHm2*	3	LOC_Os03g51580
	*HvHLHm3*	4H	contig_2546776	14.43	MLOC_36423	6	Y	*OsHLHm3*	10	LOC_Os10g01530
	*HvHLHm4*	4H	contig_53151	98.84	MLOC_67483	1	N	*OsHLHm4*	12	LOC_Os12g43620
NAM, ATAF1,2, and CUC2 (NAC)	*HvNAC1*	4H	contig_54520	51.4 (O)	MLOC_68284	1	Y	*OsNAC006*	3	LOC_Os03g42630
	*HvNAC2*	7H	contig_170782	110.27	MLOC_25708	2	N	*OsNAC5*	8	LOC_Os08g10080
	*HvNAC3*	5H	contig_54346	80.34	MLOC_68185	2	N	*OsNAC6*	6	LOC_Os06g46270
	*HvNAC4*	5H	contig_2547787	150.07	MLOC_37104	2	Y	*OsNAC9/SNAC1*	3	LOC_Os03g60080
	*HvNAC5*	7H	contig_38602	110.27	MLOC_53744	1	Y	*OsNAC10*	11	LOC_Os11g03300
	*HvNAM1*	6H	contig_1574297	53.6	MLOC_16728^L^	3	N	*OsNAC010/NAM*	7	LOC_Os7g37920
	*HvNAM2*	2H	contig_141206	57.08	MLOC_8116	1	Y			
Aberrant panicle organization	*HvAPO1*	na	contig_692	na	MLOC_75864	1	N	*OsAPO1/FBX202*	6	LOC_Os06g45460
	*HvFBX94*	5H	contig_2547870	44.24	MLOC_37150	2	Y	*OsFBX94*	3	LOC_Os03g28130
	*HvFBX258*	2H	contig_37898	54.25	MLOC_52901	1	Y	*OsFBX258*	7	LOC_Os07g42590
**TRANSPORTER GENES**										
Nitrate transporter 2 (high affinity)	*HvNRT2.1*	3H	contig_67100	55.81	MLOC_75087	2	N	*OsNRT2.1*	2	LOC_Os02g02170
	*HvNRT2.2*	6H	contig_42664	13.67	MLOC_58437	1	N	*OsNRT2.2*	2	LOC_Os02g02190
	*HvNRT2.3*	6H	contig_42664	13.67	MLOC_58438	1	N	*OsNRT2.3a*	1	LOC_Os01g50820
	*HvNRT2.4*	6H	contig_37664	13.67	MLOC_52621	1	N	*OsNRT2.3b*	1	LOC_Os01g50820
	*HvNRT2.5*	6H	contig_49761	13.67	MLOC_65110	1	N	*OsNRT2.4*	1	LOC_Os01g36720
	*HvNRT2.6*	6H	contig_114886	13.52	MLOC_1673	1	Y			
	*HvNRT2.7*	7H	contig_58466	95.25	MLOC_70747	1	N			
NRT2 partner protein (NAR2)	*HvNAR2.1*	6H	contig_127434	54.96	MLOC_3053	1	N	*OsNAR2.1*	2	LOC_Os02g38230
	*HvNAR2.2*	5H	contig_64422	155.56	MLOC_73802	1	N	*OsNAR2.2*	4	LOC_Os04g40410
	*HvNAR2.3*	6H	contig_44268	55.38	MLOC_60308	1	N			
Ammonium transporter	*HvAMT1.1*	6H	contig_240647	55.38	MLOC_33834	1	Y	*OsAMT1.1*	4	LOC_Os04g43070
	*HvAMT1.2*	2H	contig_45766	67.49	MLOC_61695	1	N	*OsAMT1.2*	2	LOC_Os02g40710
								*OsAMT1.3*	2	LOC_Os02g40730
Lysine histidine transporter	*HVLHT1*	7H	contig_85053	52.27	MLOC_79443	1	Y	*OsLHT1*	12	LOC_Os12g14100
	*HVLHT2*	7H	contig_1574246	70.54	MLOC_16705	3	Y	*OsLHT2*	8	LOC_Os08g03350
	*HVLHT3*	7H	contig_38837	70.54	MLOC_54046	4	Y	*OsLHT3*	5	LOC_Os05g14820
**OTHER GENES**										
Cytokinin oxidase/dehydrogenase (CKX)	*HvCKX1*	3H	contig_95597	46.1	MLOC_81291	1	Y	*OsCKX2/Gn1a*	1	LOC_Os01g10110
	*HvCKX2*	6H	contig_1569969	55.52	MLOC_15141	2	Y	*OsCKX5*	1	LOC_Os01g56810
	*HvCKX3*	3H	contig_1573545	68.2	MLOC_16499	2	Y	*OsCKX4*	1	LOC_Os01g71310
	*HvCKX4*	3H	contig_37260	135.62	MLOC_52060^L^	6	na	*OsCKX3*	10	LOC_Os10g34230
	*HvCKX5*	1H	contig_1560205	54.39	MLOC_11021	10	N	*OsCKX1*	1	LOC_Os01g09260
	*HvCKX6*	3H	contig_42846	45.82	MLOC_58639	1	N	*OsCKX6*	2	LOC_Os02g12770
	*HvCKX7*	2H	contig_37316	74.08	MLOC_52145	3	N	*OsCKX7*	6	LOC_Os06g37500
	*HvCKX8*	3H	contig_38743	47.1	MLOC_53923	1	N	*OsCKX8*	5	LOC_Os05g31040
								*OsCKX9*	2	LOC_Os02g12780
Cytokinin biosynthesis (IPT)	*HvIPT1*	1H	contig_1567227	37.6	MLOC_14093	1	Y	OsIPT1^**^	3	LOC_Os03g24440
	*HvIPT2*	2H	contig_71263	58.05 (O)	MLOC_76403	6	Y	*OsIPT2*	3	LOC_Os03g24240
	*HvIPT3*	3H	contig_37390	52.62	MLOC_52237^L^	1	na	*OsIPT3*	5	LOC_Os05g24660
	*HvIPT4*	3H	contig_37390	52.62	MLOC_52238	1	N	*OsIPT4*	3	LOC_Os03g59570
	*HvIPT5*	1H	contig_8161	107.29	MLOC_78718^L^	1	na	*OsIPT5*	7	LOC_Os07g11050
Cell wall invertase	*HvCIN1*	4H	contig_49313	111.22	MLOC_64782	3	Y	*OsCIN1*	2	LOC_Os02g33110
	*HvCIN2*	2H	contig_41327	58.78	MLOC_56998	4	N	*GIF1/OsCIN2*	4	LOC_Os04g33740
	*HvCIN3*	1H	contig_136454	117.49	MLOC_5612	5	Y	*OsCIN3*	4	LOC_Os04g33720
Stay-green protein	*HvSGR1*	5H	contig_53834	98.13	MLOC_67884	3	Y	*OsSGR1*	9	LOC_Os09g36200
Ferredoxin NADP(H) reductase	*HvFNR1*	7H	contig_58048	1.63	MLOC_70480	1	Y	*OsFNR1*	7	LOC_Os07g05400
	*HvFNR2*	5H	contig_138165	136.11	MLOC_6838	1	N	*OsFNR2*	3	LOC_Os03g57120
	*HvFNR3*	6H	contig_60084	3.75	MLOC_71570	2	Y	*OsFNR3*	6	LOC_Os06g01850

*na, not available*.

L *, Low confidence genes from IPK Barley Blast Server*;

*O, Oregon Wolfe*,

* *6 members*;

** *8 members*.

#### Signaling

Among the signaling gene family, HvDEP1 is the γ-subunit of G-protein and only one homolog was identified in barley (MLOC_52150), but with a low identity with rice. A rice gene, *SMG1* (*small grain1*) encodes a mitogen-activated protein kinase kinase 4 (MKK4; Duan et al., 2014). Two barley isogenes (*HvSMG1* and *HvSMG2*) were found both on chromosome 2H. In plants, the SnRK (Sucrose non-fermenting-1 Related Kinases) family includes diverse members. Both the rice (*SnRK2.1-2.9*) and barley (*HvPKABA1-9*) genomes encode nine members of the SnRK2 and SnRK1 subfamilies, respectively. Additional putative signaling gene characterized to affect NUE in rice is *ENOD93* (Early Nodulin-like protein 93; Bi et al., 2009). All six members of this family are closely linked on chromosome 6 in rice, but in barley, only three members were identified and mapped to different chromosomes (6H and 7H; see Table 2).

#### Amino acid biosynthesis

In this category, the alanine aminotransferase (AlaAT) gene family is divided into two sub-families: GPT and GGT (localized to peroxisomes) gene family (Liepman and Olsen, 2003). Five GPT and two GGT candidate genes were identified in barley from our BLAST searches (Table 2). Asparagine synthetase (ASN) and asparaginase (ASNase) have been reported to affect N utilization and seed yield in *Arabidopsis* (Lam et al., 2003; Ivanov et al., 2012). Two isogenes were identified for each family (Table 2). Glutamate synthase (GOGAT, glutamine oxoglutarate aminotransferase) manufactures glutamate from glutamine and α-ketoglutarate, and along with glutamine synthetase (GS), is recognized to play a pivotal role in N assimilation in photosynthetic organisms (Tobin and Yamaya, 2001). GOGAT isoenzymes (NAD(P)H- and Fd-GOGAT) catalyze the transfer of the amido N of glutamine to 2-oxoglutarate, using either NAD(P)H or ferredoxin as reductants (Tamura et al., 2011). There are five GS and two GOGAT genes identified in barley. Two GOGAT genes were mapped to the barley chromosome 2H (*HvGOGAT1*) and 3H (*HvGOGAT2*).

#### N assimilation and transporters

In plants, N can be taken up either as nitrate or ammonium directly from the soil through roots. Ammonium is moved into intracellular compartments by the ammonium transporter (AMT) and, then converted through the GS/GOGAT pathway into a variety of organic molecules such as amino acids for plant growth. The process of resulting molecules derived from ammonia via the GS/GOGAT cycle can be as part of primary N assimilation (Oaks, 1994). Two high-affinity AMT genes were previously characterized in barley (Zhao et al., 2014). These two AMT genes were further mapped on chromosome 6H (*HvAMT1.1*) and 2H (*HvAMT1.2*; Table 2). Another N uptake form, nitrate is primarily transported into the cell by nitrate transporters and, subsequently, it is converted to nitrite by nitrate reductase (NR) and reduced to ammonium by nitrite reductase (NiR). In barley, the BLAST searches resulted in the identification of two isogenes for both NR and NiR with very high identities to the homologs in rice. Two NR genes (*HvNR1* and *HvNR2*) were mapped on chromosome 6H (Table 2). The nomenclature of the nitrate transporters has evolved over time, as there were initially considered to be two types of nitrate transporters, low-affinity transporter (NRT1 or NPF, NRT/PTR Family) and high-affinity transporter (NRT2), described on the basis of affinities for nitrate uptake (Léran et al., 2015b). The search for nitrate transporter genes plus their partners leads to the identification of seven candidate members (*HvNRT2.1-2.7*) of the NRT2 family and three candidate members (*HvNAR2.1-2.3*) for NAR2 family (Table 2). In comparison with high-affinity transporters, the picture is a good deal more complex for the low-affinity transporters. When we considered the low-affinity nitrate transporter family in barley, at least 31 NRT1 (or NPF) isogenic loci were identified respective to homologs in rice (Table S1; Xia et al., 2015).

#### Transcriptional factors and other uncategorized genes

The complexity of multi gene families, even in a relatively simple diploid, is further illustrated by the example of transcriptional factors (TFs). Some of the characterized TFs have been shown to impact on grain yield and tolerance of drought-related stress (Table 1). Due to the existence of large numbers of TFs in barley genomes, only the functionally characterized TFs gene families (DOF, NFY, bHLH, NAC, and F-box) in plants were used as inputs to search for their homologs in barley (Table 2). Two members (*HvNAM-1* and *HvNAM-2*) of NAC TF family were identified in barley, of which, *NAM-1* (*Gpc-B1*) has been shown to be involved in N remobilization and NUE in wheat that were determined by GPC (Uauy et al., 2006). Among the uncategorized gene families, a number of cytokinin oxidase/dehydrogenase (CKK) and cytokinin biosynthesis isopentenyltransferase (IPT) were identified and mapped in barley genome owing to the important physiological function in leaf senescence delay, resulting in a modified N remobilization (Rubio-Wilhelmi et al., 2011). Other gene families, including cell wall invertase (CIN), stay green protein (SGP), and Fd-NAD(P)H reductase (FNR), have been implicated their involvements in the regulation of seed filling and root growth, and were also considered as candidate gene families for NUE (Table 1).

### Mining genes by expression and phylogenetic analyses

In selecting and evaluating identified genes associated with NUE, it was hypothesized that certain members of a gene family are more likely to be expressed in certain tissues, based on the specific trait of interest. Therefore, we examined the expression profiles of subset gene family members in barley using both microarray data and RNA-seq data. As physiological functions for the GPT, GGT, NTR2, and NAR2 families are currently under investigation in laboratory; these genes were then selected and analyzed their expression patterns in order to further reinforce the identity of barley NUE-associated genes. Among the five identified barley GPT family genes, *HvAlaAT1-1* (MLOC_66262) shows its expression in almost every tissue, but with the highest levels of expression in root and developing seed (Figure 2A). The expression of *HvAlaAT5-2* (MLOC_66427) seems to be only detectable in developing seeds. *HvAlaAT2-1* (MLOC_52901) and *HvAlaAT5-1* (MLOC_7150) have distinctly lower levels of expression in all tissues. *HvAlaAT2-2* (MLOC_69931) is the most highly expressed candidate GPT gene in leaf and also exhibits high levels of expression in caryopsis, consistent with the observation of functional GGT activity purified from the peroxisomes of leaf tissue in *Arabidopsis* (Liepman and Olsen, 2003). Of the two candidate GGT genes, *HvGGT1* (MLOC_57145) and *HvGGT2* (MLOC_17573) have distinct expression patterns (Figure 2A). In comparison, seven members of NRT2 and three members of NAR2 were identified in barley. Not surprisingly, the mRNAs for NRT2 and NAR2 were found dominantly expressed in root (Figure 3A). These RNA sequence data were compatible with the microarray data and similar expression patterns were observed (Figures 2B, 3B, and Table S2).

To determine the relations within members of a gene family in rice and barley, we performed a phylogenetic analysis to understand the evolutionary history for several of the gene families. The GPT and GGT gene families were clustered into two clades. The five putative GPT genes cluster closely (Figure 2C). The BLAST searches identified a putative GPT in rice, *OsAlaAT3-1* (LOC_Os07g01760), which is 95% identical to *HvAlaAT2-1*. Among GGT family, *HvGGT1* and *HvGGT2* were identified based on protein sequence identity to the characterized GGT in rice and cluster in a distinct clade from the GPTs with good bootstrap support (Figure 2C). The NRT2 and NAR2 families also clustered into two distinct clades (Figure 3C). Interestingly, a duplication event within the NAR2 gene family occurred in barley between the members *HvNRT2.2* (MLOC_58437) and *HvNRT2.3* (MLOC_58438).

### Integrating QTLs that may segregate with NUE genes

To evaluate NUE-associated gene(s) that may segregate with the QTLs for NUE prompts us to further examine these genes relevance to NUE phenotype. Based on the selection criteria, 10 independent mapping studies were screened. Selected field studies were carried with different parental genotypes, population size and type, locations, environments, and years (Table S4). Using RILs, several QTLs for NUE (ΔN and NHI) were identified (Mickelson et al., 2003; Kindu et al., 2014). A number of QTLs involved in YLD were mapped on several chromosomes by using segregating populations (Mansour et al., 2013). Seven of studies showed that genome wide association (GWA) mapping approach was used to look insight QTLs involved in NUE-related traits (GPC, YLD, and GW) in barley (Comadran et al., 2011; Pasam et al., 2012; Varshney et al., 2012; Wang et al., 2012a; Berger et al., 2013; Pauli et al., 2014; Mohammadi et al., 2015). Most recent advance on mapping GPC trait showed that a number of novel marker-trait associations were made using GWA study on U.S. barley breeding populations and some QTLs were mapped, along with several other loci that affect YLD (Pauli et al., 2014; Mohammadi et al., 2015). As two publications lack consensus markers on their maps, it is a challenge to track and normalize the marker location and specifically compare these QTLs within the consensus map (Mickelson et al., 2003; Kindu et al., 2014).

**Figure 1:**
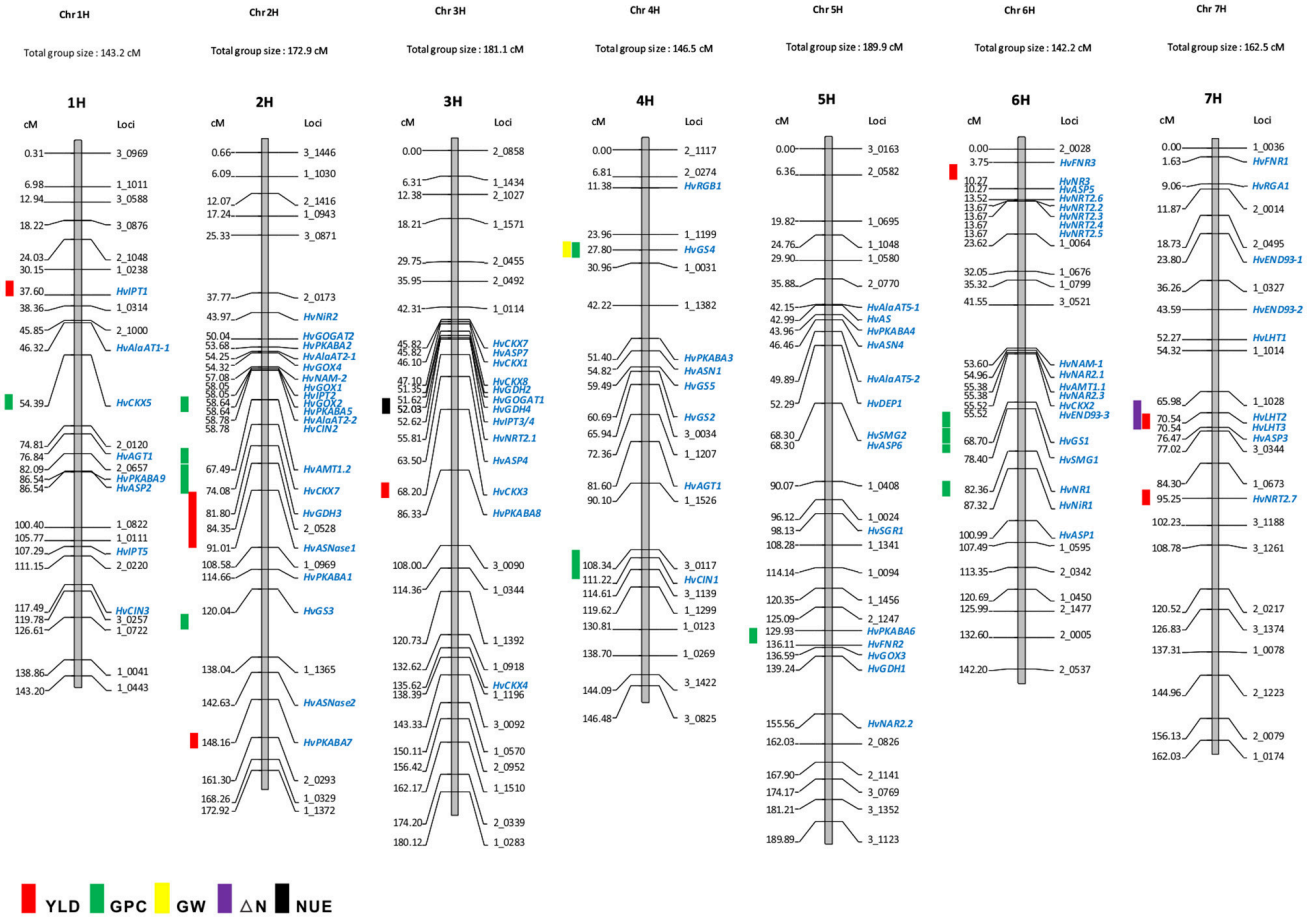
**Co-localization of NUE-associated genes and QTLs for NUE on barley consensus genetic map**. The QTLs for NUE-related traits were marked on the left side of the chromosome based on the marker location (cM). The genetic map shows the position of NUE candidate genes as mapped in the “Morex” × “Barke” population (Mayer et al., 2012). Some mapped SNP markers are shown above. The list of genetic locations for the barley NUE candidate genes is shown in Table 2.

Owing to the technique difficulties of conducting a meta-analysis of QTLs for NUE using GWA studies, we only performed a comparison to search for the co-segregation between identified genes and QTLs for NUE-related traits in barley consensus map. The NUE-associated genes were then projected on the barley consensus map where QTLs co-localized with candidate genes were marked in different colors (Figure 1). In Table 3, a number of QTLs were listed for GPC traits. Two of them, 6H (45.4 cM) and 2H (53.53 cM) were shown to be close with several gene clusters, including clustered *HvNAM-1* and *HvNAM-2* (Pauli et al., 2014). Other two QTLs from 4H (26.2 cM) and 5H (137.2 cM) were shown their locations in the vicinity of *HvGS4* and a gene cluster, including *HvPKABA4, HvFNR2*, and *HvGOX3* (Pauli et al., 2014). In addition, two genes for N assimilation, *HvNR1* and *HvNiR2* were identified to be close to the mapped GPC trait on 6H (83.89 cM; Pasam et al., 2012). *HvGS4* was also recognized and would be in correlation with GPC trait on 4H (27.75 cM; Mohammadi et al., 2015) and GW trait on 4H (26.2 cM; Wang et al., 2012a). Two cytokinin oxidase/dehydrogenase genes, *HvCKX5* and *HvCKX7* appear to be associated with GPC trait on 1H (55.49 cM; Pasam et al., 2012) and YLD trait on 2H (54.1 cM), respectively (Mansour et al., 2013). Pauli et al. (2014) revealed that one of two QTLs mapped for YLD traits is on 3H (55.6 cM) where more than 10 NUE-associated genes were clustered and interestingly, this QTL is close to the previously reported orthologous QTL for NUE (Quraishi et al., 2011). A search of this conserved region (3H) in barley showed that homologous gene loci were identified. Including *HvGOGAT1*, at least 16 annotated genes would be responsible for NUE regulation (Table S3). Another one QTL mapped for YLD trait is localized to 2H (132.48 cM) where a kinase gene, *HvPKABA7* was determined (Pauli et al., 2014). Additional YLD trait on 6H (7.87–8.74 cM) was mapped close to a gene cluster comprised of *HvNR2, HvASP5*, and five high-affinity nitrate transporters (*NRT2.2–2.6*; Berger et al., 2013). The QTLs for traits of ΔN (64.18–70.68 cM, 7H) and YLD (64.98 cM) on 7H would correlate with two TF genes, *HvLHT2* and *HvLHT3* (Mickelson et al., 2003; Comadran et al., 2011). Unfortunately, neither of QTLs for NHI and NUE was observed potential correlations with NUE-associated genes in barley consensus map (Table 3).

**Table 3:** **Analysis of genes that may be associated with QTLs for NUE**.

**Marker**	**Chr**	**Interval (cM)**	**Consensus map (cM)**	**QTL**	**Genes co-localized**	**Trait**	**LOD**	**Mean variation (*R*^2^)**	**References**
GBS0469	1H	133.00	139.86	QYld-1H.133		YLD	na	3.80	Varshney et al., 2012
**11_10275**–**11_10597**	1H	42.52–44.6	35.77–36.64	QYld-1H.42-44	***HvIPT1***	YLD	4.70	3.64	Mansour et al., 2013
**2_0798**	1H	55.49	55.49	QGpc-1H.55	***HvAlaAT1-1, HvCKX5***	GPC	na	0.78	Pasam et al., 2012
12_30948	1H	15.76	15.91	QGpc-1H.16		GPC	na	na	Mohammadi et al., 2015
11_10357	1H	100.69	103.99	QGw-1H.101		GW	na	3.60	Wang et al., 2012a
11_10396	1H	93.95–96.92	98.68	QGn-1H.94-97		GN	na	5.90	
**hvbkasi**	2H	0–18	67.22–75.23	QGpc-2H	***HvCIN2, HvAMT1.2, HvCKX7***	GPC	8.66	26.30	Mickelson et al., 2003
2_1304	2H	33.74	33.74	QGpc-2H.34		GPC	na	0.45	Pasam et al., 2012
**1_0685**	2H	63.53	63.53	QGpc-2H.64	***HvCIN2, HvAMT1.2***	GPC	na	0.62	
**11_11400**	2H	53.53	58.50	QGpc-2H.54	***HvNAM-2, HvGOX1, HvIPT2, HvGOX2, HvGOGAT2, HvPKABA5, HvAlaAT2-2, HvCIN2***	GPC	na	0.64	Pauli et al., 2014
11_20340	2H	85.92	98.74	QGpc-2H.86		GPC	na	1.72	
acaa210	2H	48–70	na	QYld-2H		YLD	4.13	12.30	Mickelson et al., 2003
VVLOCI	2H	128–146	na	QYld-2H		YLD	3.66	11.00	
**11_10191**	2H	63.53	72.99	QYld-2H.63	***HvCKX7***	YLD	na	8.29	Comadran et al., 2011
**12_10579**	2H	132.48	149.93	QYld-2H.132	***HvPKABA7***	YLD	na	1.70	Pauli et al., 2014
**11_11430**–**11_10818**	2H	54.1–78.03	73.89–90.48	QYld-2H.54-78	***HvCKX7, HvGDH3, HvASNase1***	YLD	5.70	6.10	Mansour et al., 2013
E35M61-355–E39M55-417	2H	108.8–130.7	na	QGw/QNHI/QNUE-2H.119		GW/NHI/NUE	3.20	14.50	Kindu et al., 2014
**2_0944**	3H	122.14–130.82	122.14–130.82	QGpc-3H.122-130	***HvCKX4***	GPC	na	0.68	Pasam et al., 2012
acag155	3H	166–186	na	QYld-3H		YLD	4.56	13.40	Mickelson et al., 2003
acgc469	3H	324–340	na	QYld-3H		YLD	7.18	20.30	
bPb_4616	3H	153.00	103.59	QYld/BY-3H.153		YLD	na	1.80	Varshney et al., 2012
**12_31010**	3H	55.57	67.86	QYld-3H.52	***HvASP4, HvCKX3***	YLD	na	1.45	Pauli et al., 2014
E38M50-242–E38M54-158	3H	123.2–126.4	98.4–125.4	QNUE-3H.125-133		NUE	6.84	20.80	Kindu et al., 2014
**2_0515**	4H	97.06–108.70	97.06–108.70	QGpc-4H.97-108	***HvCIN1***	GPC	na	0.87	Pasam et al., 2012
**11_21070**	4H	26.19	28.00	QGpc-4H.26	***HvGS4***	GPC	na	0.74	Pauli et al., 2014
**11_20302**	4H	27.75	28.00	QGpc-4H.28	***HvGS4***	GPC	na	na	Mohammadi et al., 2015
**11_20606**	4H	26.19	28.00	QGw-4H.26	***HvGS4***	GW	na	7.80	Wang et al., 2012a
11_20013	4H	123.29	146.48	QGn-4H.123		GN	na	4.50	
1_0871	5H	110.26	110.26	QGpc-5H.110		GPC	na	0.73	Pasam et al., 2012
3_1417	5H	96.10	96.10	QGpc-5H.96		GPC	na	0.15	Berger et al., 2013
12_20770	5H	42.32	35.88	QGpc-5H.42		GPC	na	1.27	Pauli et al., 2014
**11_10095**	5H	137.16	132.32	QGpc-5H.137	***HvPKABA4, HvFNR2, HvGOX3***	GPC	na	1.92	
11_10254	5H	179.06	169.70	QGpc-5H.177-180		GPC	na	1.28	
actc410	5H	142–174	na	QYld-5H		YLD	4.05	12.00	Mickelson et al., 2003
VrnH1	5H	14.80	181.60	QYld-5H.14		YLD	6.90	14.26	Mansour et al., 2013
11_20553	5H	2.81	1.91	QGn-5H.3		GN	na	6.60	Wang et al., 2012a
**HVM74/12_10199**	6H	250–256	58.71–61.10	QGpc-6H.250-256	***HvNAM-1, HvNAR2.1, HvAMT1.1***	GPC	19.50	45.90	Mickelson et al., 2003
**1_1147**	6H	83.89	83.89	QGpc-6H.84	***HvNR1***	GPC	na	0.91	Pasam et al., 2012
2_0537	6H	142.20	142.20	QGpc-6H.142		GPC	na	0.13	Berger et al., 2013
**12_10199**	6H	45.40	49.67	QGpc-6H.45	***HvNAM-1***	GPC	na	2.12	Pauli et al., 2014
**12_10199**	6H	49.23	49.67	QGpc-6H.49	***HvNAM-1***	GPC	na	na	Mohammadi et al., 2015
**12_11353**	6H	55.55	56.06	QGpc-6H.55	***HvNAM-1, HvNAR2.1, HvAMT1.1,***	GPC	na	na	
11_10954	6H	58.72	59.25	QGpc-6H.59		GPC	na	na	
**12_31003**	6H	64.07	64.65	QGpc-6H.64	***HvGS1***	GPC	na	na	
**12_30346**	6H	65.24	65.83	QGpc-6H.65	***HvGS1***	GPC	na	na	
**3_0651**–**2_1204**	6H	7.87–8.74	7.87–8.74	QYld-6H.7-8	***HvNR3, HvASP5***	YLD	na	0.03	Berger et al., 2013
actt166	6H	214–226	na	QYld-6H		YLD	4.85	14.20	Mickelson et al., 2003
acgc132	6H	68–90	na	QΔN-6H		ΔN	4.70	13.90	
2_0245	7H	12.42	12.42	QGpc-7H.12		GPC	na	0.51	Pasam et al., 2012
2_0570	7H	112.46	112.46	QGpc-2H.41		GPC	na	0.49	
2_0217	7H	121.09	121.09	QGpc-2H.42		GPC	na	1.57	
12_31199	7H	86.40	88.06	QGpc-7H.86		GPC	na	1.43	Pauli et al., 2014
11_21209	7H	129.91	130.64	QGpc-7H.130		GPC	na	1.14	
acaa327	7H	238–244	na	QYld-7H		YLD	4.40	13.20	Mickelson et al., 2003
HVM5	7H	216–254	141.33–157.75	QYld-1H		YLD	3.59	10.80	
**11_11348**	7H	70.40	64.98	QYld-7H.70	***HvLHT2, HvLHT3***	YLD	na	10.99	Comadran et al., 2011
11_11445	7H	84.92	85.28	QYld-7H.84		YLD	na	10.46	
**2_0685**	7H	94.34	94.34	QYld-7H.94	***HvNRT2.7***	YLD	na	0.05	Berger et al., 2013
11_10327–11_20074	7H	54.37–58.2	36.36–38.28	QYld-7H.54-58		YLD	5.40	6.02	Mansour et al., 2013
acgc140	7H	32–46	na	QΔN-5H		ΔN	4.26	12.70	Mickelson et al., 2003
**pinb1**	7H	52–64	64.18–70.68	QΔN-7H.52-64	***HvLHT2, HvLHT3***	ΔN	3.59	10.80	

*GPC, grain protein content in grains; YLD, amount of a crop of per available unit; GN, nitrogen content in grains; ΔN, N remobilization efficiency (changes of leaf N between anthesis and maturity); NIH, N harvest index); NUE (grain yield/total amount of nitrogen supply; GW, average weight of a cereal as measured in pounds per bushel). QTLs co-segregated with NUE-associated genes are marked in bold*.

## Discussion

### Identification of genes that may affect NUE

We provide a detailed analysis of candidate genes associated with NUE. To our knowledge, most of NUE-associated genes identified in this paper are not published. The full sequences for all genes can be easily accessible on the basis of the gene ID listed in Table 2. However, barley possesses a relative large and highly repetitive genome (5.1 Gb) that has slowed the processing of a complete sequence with fine structure and high resolution (Mayer et al., 2012). Exploration of the new regenerated partial (~1.7 Gb) genomic sequence of Morex barley genes revealed that not only distal ends of chromosomes contain most of the gene-enriched BACs with high recombination rates, but also gene-dense regions with suppressed recombination (Muñoz-Amatriaín et al., 2015). This might be explained by the findings that some of the identified genes (e.g., *HvASNase1* and *HvASNase2*) were assembled with more than one gene model and some of them (e.g., *HvDEP1* and *HvAlaAT1-1*) exhibit sequences of low confidence but with high identities with homologs in rice (Table 2).

A number of genes of primary N uptake and assimilation have been targeted as bioengineering candidates to attempt and increase the NUE of crop plants. Manipulating one or more of these gene products is expected to potentially increase the NUE of crops and therefore, it is important to initially understand the genetic components that contribute to these processes. Recently, a γ-subunit of heterotrimeric G protein (DEP1) was reported to regulate NUE in rice by improving N Uptake and assimilation that result in NHI and YLD under a moderate input of N fertilizer (Sun et al., 2014). This research underpinned the signaling role of nitrogen-heterotrimeric G protein in the nutrient regulation of plant development and uncovered a potential new strategy for environmentally sustainable agriculture by NUE. In barley, three subunits (*HvDEP1, HvRGA1*, and *HvRGB1*) of heterotrimeric G protein were identified; however, their signaling roles in regulating NUE are remained to be unraveled. Same as G protein, actions in seed size regulation for the new identified MKK (*HvSMG1* and *HvSMG2*) are also largely unknown. Another protein kinase, SnRKs play a key role at the interface between metabolic and stress signaling, suggesting their potentialities for the manipulation to improve crop performance in critical environments (Coello et al., 2011). Overexpression of a *SnRK1* in tomato elevates carbon assimilation and N uptake that resulted in influences of fruit development (Wang et al., 2012b). In comparison with a recent report (Seiler et al., 2014), a more complete information was given here for the nine identified SnRK2 genes (*HvPKABA1–9*). To date, only *HvPKABA1* has been functionally characterized to play as an intermediate in suppressing GA-inducible gene expression in barley aleurone layers (Gómez-Cadenas et al., 1999).

Including amino acid biosynthesis (e.g., GOGAT and GS) and N transporters (NRT and AMT), such gene families have been well-studied owning to the central players in driving NUE-related traits in major crops (Quraishi et al., 2011; Beatty et al., 2013). In higher plants GOGAT occurs as two antigenically distinct forms (Fd-GOGAT and NAD(P)H-GOGAT) with differences in protein size, tissue localizations, and physiological functions (Esposito et al., 2005). NAD(P)H-GOGAT is controlled by the N-status in response to N and therefore, it was postulated to play a fundamental role in primary N assimilation in plants (Vanoni and Curti, 1999). A recent study showed that deletion of the *OsNADH-GOGAT2* gene in rice caused remarkable reductions in yield and plant biomass (Tamura et al., 2011). Based on protein molecular weight, we verified that *HvGOGAT1* (3H) is *NAD(P)H-GOGAT*, and another isogene, *HvGOGAT1*(2H) is *Fd-GOGAT* respective to three homologs in rice (Table 2). GS and ASN are also two key enzymes involved in ammonium assimilation and their roles in nitrogen remobilization and NUE have been elucidated (Lam et al., 2003; Brauer et al., 2011). Five members (*HvGS1-5*) of GS gene family were identified and their gene profiles were further supported by a recent study (Avila-Ospina et al., 2015). Interestingly, bioengineered genes that have shown an NUE phenotype in crops are not primary N assimilation genes, but instead are genes involved in N metabolism further downstream than GOGAT and GS, such as AlaAT and TFs. One (MLOC_66262) of five identified *HvAlaAT* has been manipulated by ectopic expression to enhance NUE and biomass in crops, confirming its role in NUpE and storage (Shrawat et al., 2008). Likewise, other biotechnology example of improvement in N uptake is to increase the efficiency of N-related transporters (Good et al., 2004). Recent reports revealed that nitrate transporter affects nitrogen accumulation in *Arabidopsis* embryo and in addition, over-expression of low affinity transporter, *OsPTR6* in rice resulted in an increase of plant growth (Fan et al., 2014; Léran et al., 2015a). Four additional barley members (*NRT2.4–2.7*) of NRT2 were identified and most of them have not been investigated previously (Table 2). We also showed the presence of at least 31 members of NRT1/ NPF in barley genome (Table S1); however, none of them has been functionally characterized, suggesting the future challenges of investigating their multiple roles for N transport within a large gene family. Notably, N uptake by transporters depends on appropriate carbon skeletons to allow for the synthesis of the different transported compounds. Thus, simply up-regulating these N-related transporters would not necessarily increase NUE in plants (Hawkesford, 2012). This would be explained by the finding of two-component system, including NRT2 with a partner protein (NAR2), for a functional nitrate transport in *Arabidopsis* and crops (Tong et al., 2005; Orsel et al., 2006; Yan et al., 2011).

A recent survey showed that the rice contains more than 2000 TFs distributed in 63 families, based on the conserved DNA-binding domains and structural hallmarks (Gao et al., 2006). Logically, a large number of TFs would be integrated in barley genome. To date, only a few of TFs have been attempted for unraveling their potential physiological actions in affecting YLD or in improving N assimilation, N remobilization, and abiotic stress-related resistance in plants (Uauy et al., 2006; Terao et al., 2010; Li et al., 2013; Chiasson et al., 2014; Chen et al., 2015; Yang et al., 2016). These researches revealed substantial functions of TFs in orchestrating N metabolism and transport processes. However, there is still a long way to go for identifying essential TFs within a gigantic genome and concurrently, unveiling their crucial roles in accurately deploying specific genes for NUE in plant growth and development.

### Analysis of gene expression patterns

**Figure 2:**
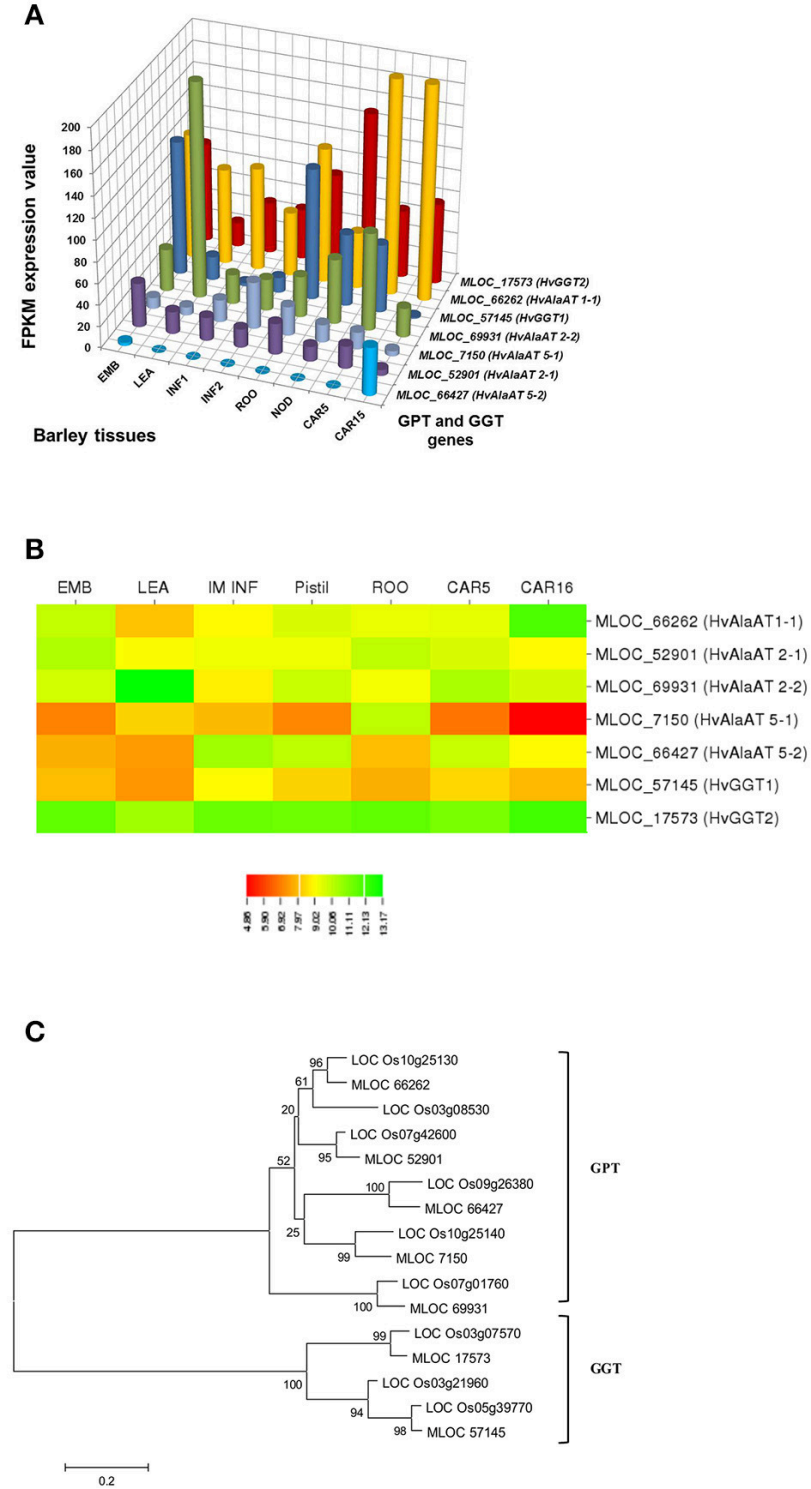
**Expression profiles of the GPT and GGT in different tissues of barley (A,B) and phylogenetic analysis of these genes in rice and barley (C)**. The RNA sequence data for GPT and AGT genes includes three biological replicates per tissue. The results are given in FPKM expression values for RNA_seq. Microarray data value is Log10 intensity and MAS 5.0 normalization. Phylogenetic tree of 16 members of GPT and GGT gene family from rice and barley were conducted by MEGA 6, using Neighbor-Joining method by MUSCLE alignment.

**Figure 3:**
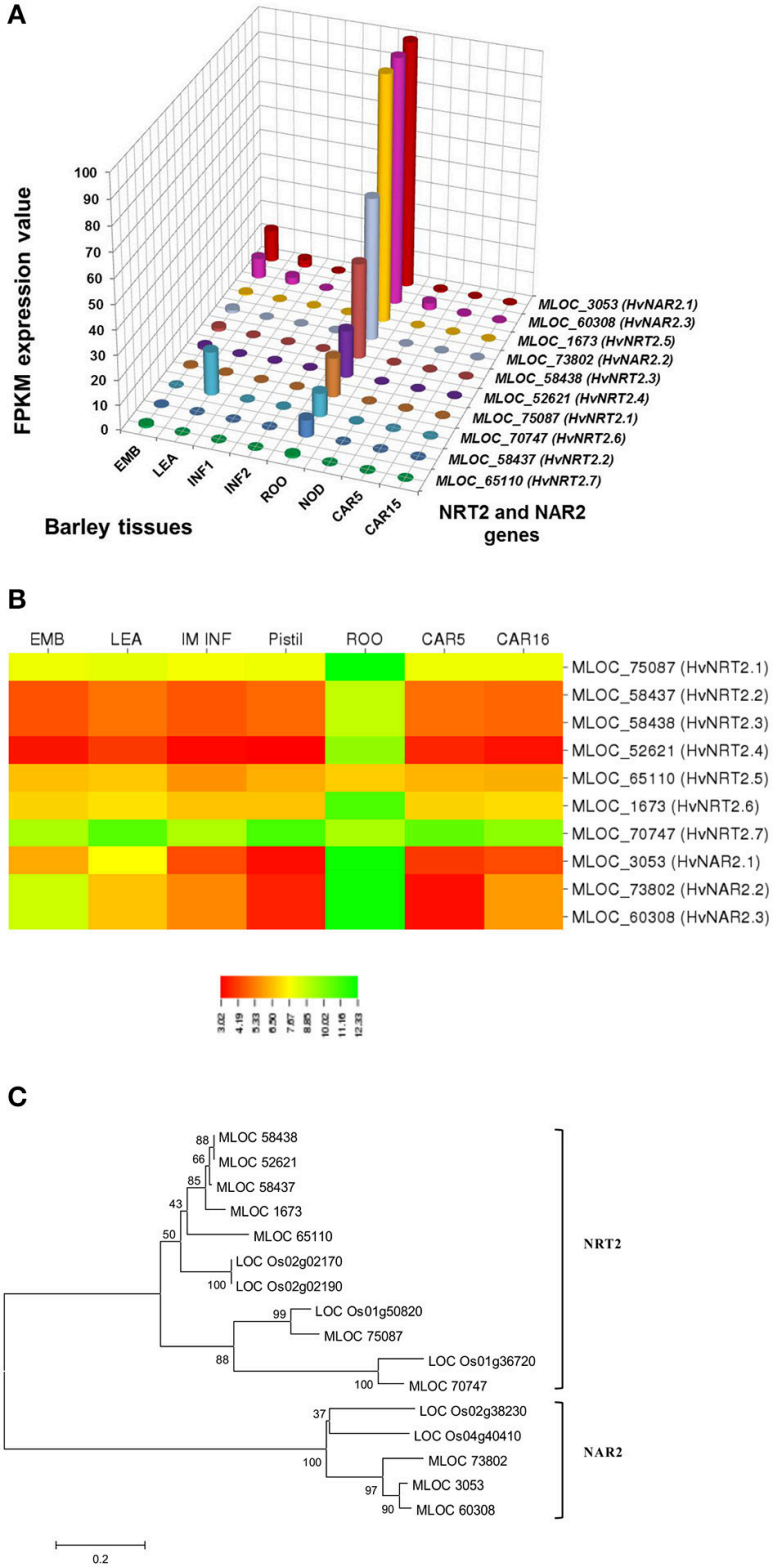
**Expression profiles of the NRT2 and NAR2 in different tissues of barley (A,B) and phylogenetic analysis of these genes in rice and barley (C)**. The RNA sequence data for NRT2 and NAR2 genes includes three biological replicates per tissue. The results are given in FPKM expression values for RNA_seq. Microarray data value is Log10 intensity and MAS 5.0 normalization. Phylogenetic tree of 16 members of NRT2 and NAR2 gene family from rice and barley were conducted by MEGA 6, using Neighbor-Joining method by MUSCLE alignment.

One approach to understand how plants respond to N is to analyze gene expression using transcription profiling technology (Nunes-Nesi et al., 2010). To evaluate whether a gene may be a candidate that is involved in a particular process, one of the tools available is the tissue-specific gene expression patterns. We used the RNA-seq data from the GPT, GGT, NRT2, and NAR2 gene families (Figures 2A, 3A) to depict that the tissue-specific expression of candidate genes matched their proposed functions. For example, MLOC_66427 is predominantly expressed during caryopsis and shows little expression in roots, whereas MLOC_66262 is highly expressed in roots, matching the proposed function of MLOC_66262 as being involved in alanine biosynthesis in this tissue. Therefore, knockouts of MLOC_66262 are expected to have a greater impact on N uptake and transport to the shoots than other GPT candidate genes. Similarly, the analysis of the NRT2 and NAR2 genes demonstrated that several of the genes are expressed almost entirely in roots, while others are expressed predominantly in leaves (Figure 3A). Thus, it would be logical to target those genes expressed in roots to determine their effects on N sensing and uptake.

We further analyzed the expression patterns of these genes using different databases that contain barley microarray expression data (http://www.plexdb.org/plex.php?database=Barley), which is compatible with RNA_seq data (Figures 2B, 3B, and Table S2). However, we found that the RNA-seq data provided greater discrimination between genes, particularly when examining the effect of a treatment on genes with low expression levels and genes with high identities. This phenomenon would ascribe to the lack of specificity for probe targeting a specific gene in microarray analysis. However, to confirm the role, which candidate genes play in a particular phenotype, the predictions that arise from examining the tissue-specific expression data stress the importance of detailed genetic experiments under the correct conditions. The further evolutionary analyses of a number of gene sequences and locations in rice and barley suggested that they may evolve as a result of tandem duplications (Figures 2C, 3C). The tight linkage of these gene duplications would make it difficult to determine which gene is more important and should a NUE QTL be identified as co-segregating with these genes.

### Co-localization of NUE-associated genes and QTLs for NUE

Early researches on QTL-mapping for NUE in cereals showed that NUE traits are regulated by some conserved key gene clusters among cereal genomes, suggesting that the evolutionarily conserved regions exist for NUE within the genomes of cereals (Quraishi et al., 2011; Liu et al., 2012). The presences of conserved structure and function for key genes in major crops, allow us to examine the proposed a correlation between GOGAT, GS and NUE in barley. *HvGOGAT1* (MLOC_13604) displayed the highest identity respective to OsGOGAT1 (LOC_Os01g48960) and was mapped to 3H (51.6 cM). A number of candidate genes were identified and clustered along with this conserved region (Figure 1 and Table S3). Interestingly, we characterized that one mapped YLD trait (55.6 cM on 3H) may be in correlation with *HvGOGAT1* between 45.82 cM and 55.81 cM on 3H (Figure 1). Another member, *HvGOGAT2* (MLOC_13604) was mapped to the location of 2H (50.03 cM), assembling with more than 10 NUE-associated genes, including *HvPKABA2/5, HvAlAaT2-1/2, HvNAM-2, HvGOX1/2/4*, and *HvIPT2* (Table 2). Three QTLs for YLD and GPC were analyzed to be pooled on 2H, indicating a potential correlation between the NUE-associated genes and NUE-related traits in this region (Mickelson et al., 2003; Pasam et al., 2012; Pauli et al., 2014). As the majority of dicots with increased GS activity is consistent with high biomass accumulation, particularly under less N conditions. The most used strategy of bioengineering NUE by far is through the modification of GS (Brauer and Shelp, 2010). We found that two (*HvGS1* and *HvGS4*) of five GS genes would correlate with both GPC and GW traits, which are on the locations of 6H (64.65–65.83 cM) and 4H (28.0 cM), but probably there is no correlation with the mapped YLD trait (Wang et al., 2012a; Pauli et al., 2014; Mohammadi et al., 2015). The projection of all NUE-associated genes on the barley consensus map revealed that a number of gene loci were clustered, including regions of 2H (43.97–67.49 cM), 3H (45.83–86.33 cM), 5H (42.15–68.30 cM), and 6H (3.75–23.62 cM; 53.6–87.32 cM). Every gene cluster contains one or more N-associated transporters and a variety of TFs (Figure 1). This phenomenon suggests these NUE-associated genes potential roles in the regulation of NUE-related traits in barley.

An illustration of gene(s) that would correlate with QTLs for NUE-related traits will help to track these genes when genomic selection is considered. The marker could be used for map-based cloning of a gene that affects N uptake and utility (Sun et al., 2014). However, the correlations between NUE-associated genes and QTLs for NUE are significantly affected by the quality of mapping studies and some potential factors (see below). Therefore, it is not surprising that the comparison between identified genes and selected QTLs resulted that a small number of NUE-associated genes are co-localized with the QTLs for NUE-related traits (Table 3). Moreover, given the presence of a large number of TFs, nitrate transporters, and uncategorized genes that are not identified in barley genome, we cannot exclude that there would have many additional candidate genes that correlate with particular traits for NUE.

### Challenges of evaluating traits and genotyping for NUE

Breeding efforts to enhance the NUE of crops need to be specifically targeted to improve NUE. However, owing to the complexity of phenotypic and physiological traits, there are no standard traits for evaluating NUE. Therefore, several NUE-related traits are selected and considered to affect NUE (Table 3). The first QTL map of barley was developed and YLD was the only trait relevant to NUE that was studied (Hayes et al., 1993). Recent report in maize showed that GN and NHI are the two of important traits related with NUE (Li et al., 2015). Measuring NUE is a significant challenge because it is technically difficult to determine the N content of soil, of different tissue types, and also the N content can be highly variable between genotypes and environments (Han et al., 2015). The analysis of field trial data is also complicated when the variability in both the level of available N and the year-to-year variation are integrated. Theoretical studies and computer simulations have demonstrated that estimates of the proportion of genotypic variance explained by a QTL, especially for small samples, are often inflated, regardless of the statistical method used (Allison et al., 2002). Particularly, the weights given to individual marker-trait associations as components of selection indices can be severely biased and the prospect of MAS overestimated. The number and quality of QTL detected in a given study depends on several critical factors, including the size and type of the population, the traits, the environments, and the genome coverage of markers. It is common for QTL to vary, depending on the test environment that QTL found in 1 year will be different from those in the next year, even when using the same testing location. Besides, genes with major effects can be studied by segregation analysis; the numerous genes that have minor effects on NUE traits are much more challenging to identify since they usually cannot be investigated individually, although QTLs with minor effect could be detected by increasing the population size. Finally, a significant amount of genetic variation impact on phenotyping a QTL, as it is for traits such as NUE is unlikely to involve major genes or QTLs, but rather a number of loci with moderate effects, and a number of loci with minor effects that synergistically contribute to the traits (Byers, 2005).

Some key factors that lead to changes in the NUE components and impacts on phenotyping, QTL mapping, and selecting candidate genes for NUE improvement are still the challenges for current crop breeding (Han et al., 2015). Nevertheless, some of instructions are pointed out here to be considered for future conducting experiments. (1) Consensus markers should be preferentially applied such that the specific QTLs can be placed on a consensus genetic map. In several of the studies, it was difficult to determine an accurate map location (Mickelson et al., 2003; Kindu et al., 2014). This indicates that the use of a consensus genetic map as well as increases of marker density will facilitate the identification of specific genes for a particular trait, which will be a benefit to both traditional breeding and biotechnological approaches. (2) In order to understand thoroughly this type of difference in the field, multiple field trials with large numbers of plots should be performed (Rothstein et al., 2014). Moreover, field trials need to be done over multiple growth years as well as increasing population size to assure the accuracy of mapping studies. (3) Field data should be collected on the level of available N. Researchers normally use a few N measurements for an entire field site, and then merge the measurements to come up with a single number for available N for an entire trial site. However, the level of available N varies over very small distances and therefore, if N uptake is being measured, the researcher needs to know what N was available in the soil as well in order to accurately assess the phenotypes and make conclusions about QTLs and other markers in field studies.

## Conclusion

Over-use of N fertilizer gives rise to environmental issues in modern agriculture. The mining of favorable gene variants for NUE is a fundamental strategy to tackle these negative effects (Chao and Lin, 2015). A comprehensive overview of gene structure and basic function in N assimilation, transport and metabolism is central to modern plant biology, both with respect to breeding and engineering crop plants for desirable traits. We have identified and mapped a number of the NUE-associated genes. Some of them co-segregate with field evaluated QTLs, but many do not. We have also emphasized that there are very few ideal QTL studies that have measured NUE in the field at multiple years and in addition, some potential cues may result in a restraint of this process. This study, with the genes of interest being placed on a consensus genetic map, will contribute to a more thorough study of their physiological significances on NUE regulation in barley and, in the future provide a framework for a similar genetic analysis in the more complex cereals, such as wheat.

## Author contributions

MH, JW, and TS conducted collection and analysis of all data and TS prepared the initial draft of manuscript. PB did much of work on assisting with the gene identification and editing the manuscript. TS and AG developed the concept and were responsible for approving the final draft of the manuscript. All authors reviewed the manuscript.

## Funding

This work was supported by the grant of Sciences and Engineering Research Council of Canada (RES0001296) and Priority Academic Program Development of Jiangsu Higher Education Institutions (PAPD).

### Conflict of interest statement

The authors declare that the research was conducted in the absence of any commercial or financial relationships that could be construed as a potential conflict of interest.
